# Percutaneous angioplasty and/or stenting versus aggressive medical therapy in patients with symptomatic intracranial atherosclerotic stenosis: a 1-year follow-up study

**DOI:** 10.3389/fnagi.2023.1192681

**Published:** 2023-06-16

**Authors:** Xiaohui Li, Xiaodan Qin, Chengfang Liu, Lin Zhu, Meng Wang, Teng Jiang, Yukai Liu, Shuo Li, Hongchao Shi, Huiling Sun, Qiwen Deng, Junshan Zhou

**Affiliations:** ^1^Department of Neurology, Nanjing First Hospital, Nanjing Medical University, Nanjing, China; ^2^General Clinical Research Center, Nanjing First Hospital, Nanjing Medical University, Nanjing, China; ^3^Department of Neurology, Beijing Tiantan Hospital, Capital Medical University, Beijing, China

**Keywords:** ischemic stroke, symptomatic intracranial atherosclerotic stenosis, percutaneous transluminal angioplasty and stenting, propensity score matching, recurrent stroke

## Abstract

**Background:**

Symptomatic intracranial atherosclerotic stenosis (sICAS) is one of the common causes of ischemic stroke. However, the treatment of sICAS remains a challenge in the past with unfavorable findings. The purpose of this study was to explore the effect of stenting versus aggressive medical management on preventing recurrent stroke in patients with sICAS.

**Methods:**

We prospectively collected the clinical information of patients with sICAS who underwent percutaneous angioplasty and/or stenting (PTAS) or aggressive medical therapy from March 2020 to February 2022. Propensity score matching (PSM) was employed to ensure well-balanced characteristics of two groups. The primary outcome endpoint was defined as recurrent stroke or transient ischemic attack (TIA) within 1 year.

**Results:**

We enrolled 207 patients (51 in the PTAS and 156 in the aggressive medical groups) with sICAS. No significant difference was found between PTAS group and aggressive medical group for the risk of stroke or TIA in the same territory beyond 30 days through 6 months (*P* = 0.570) and beyond 30 days through 1 year (*P* = 0.739) except for within 30 days (*P* = 0.003). Furthermore, none showed a significant difference for disabling stroke, death and intracranial hemorrhage within 1 year. These results remain stable after adjustment. After PSM, all the outcomes have no significant difference between these two groups.

**Conclusion:**

The PTAS has similar treatment outcomes compared with aggressive medical therapy in patients with sICAS across 1-year follow-up.

## Introduction

Stroke is the second leading cause of mortality globally (Feigin, 2007). Intracranial atherosclerotic stenosis (ICAS) is one of the main causes of ischemic stroke and is closely associated with a high incidence and mortality of stroke (Gutierrez et al., 2022). Patients with symptomatic intracranial atherosclerotic stenosis (sICAS) are particularly at high risk for recurrent stroke (Wang et al., 2014). Therefore, effective treatment and secondary prevention are of great significance in reducing the high incidence of cerebrovascular events (Qureshi and Caplan, 2014).

For patients with sICAS who underwent ischemic stroke or transient ischemic attack (TIA), aggressive medical treatment should be given first. Percutaneous angioplasty and/or stenting (PTAS) can be used in sICAS patients with ineffective medical therapy (Powers et al., 2019; Turan et al., 2022). A series of studies (Derdeyn et al., 2014; Zaidat et al., 2015; Gao et al., 2022) have shown no significant benefit from PTAS compared with medical therapy alone. Another study have shown that the 30-day stroke or mortality of stenting is significantly higher than that of medical therapy, suggesting that aggressive medical therapy is superior to PTAS in patients with sICAS (Derdeyn et al., 2014). However, a number of subsequent prospective and retrospective studies have reported the benefits of endovascular therapy (Yu et al., 2012; Miao et al., 2015a,b; Gao et al., 2016; Maier et al., 2018), and individualized endovascular therapy may be effective and safe in patients with sICAS (Ma et al., 2018; Mao et al., 2022). Therefore, the optimal treatment of patients with sICAS remains elusive.

In this study, we aimed to prospectively analyze the effects of PTAS and aggressive medical therapy on sICAS patients within 1 year, and further explore their influence on ischemic events through propensity score matching (PSM) analysis.

## Materials and methods

This observational prospective cohort study was conducted at the Stroke Center of Nanjing First Hospital, Nanjing Medical University, and aimed to investigate the efficacy of PTAS vs. aggressive medical therapy in patients with sICAS. Consecutive patients were collected from an observational study of the AISRNA study (^1^NCT04175691) between March 2020 and February 2022. Our inclusion criteria were: (1) patients with ≥70% stenosis of the main trunk of the middle cerebral artery (MCA), internal carotid artery (ICA), vertebral artery (VA), or basilar artery (BA); (2) TIA or stroke in the territory of the target lesion area; and (3) modified Rankin Scale (mRS) ≤ 2. Patients with (1) a potential source of cardiac embolism, (2) mRS > 3, (3) extracranial vascular stenosis > 50%, (4) other known causes of stroke subtypes, and (5) emergency stenting were excluded. The study enrolled 207 eligible patients. The baseline characteristics, including age, sex, body mass index (BMI), medical history, low-density lipoprotein-cholesterol (LDL-C), systolic blood pressure (SBS), symptomatic qualifying artery, the degree of stenosis and National Institute of Health Stroke Scale (NIHSS) score were collected.

The patients were divided into the PTAS group and aggressive medical group both receiving the guidelines for conventional treatment, which included aspirin 100 mg plus clopidogrel 75 mg daily for 90 days (aspirin or clopidogrel alone daily thereafter) and control of stroke risk factors. The primary outcome was the recurrence of stroke or TIA in the same territory within 1 year. Adverse events included disabling stroke, symptomatic intracranial hemorrhage, or death within 1 year. All enrolled individuals were followed up through telephone or outpatient visit. They were admitted to hospital for corresponding imaging examination, included an assessment of the mRS, and establishing whether the patient had any adverse events if necessary. Disabling stroke was defined as an mRS score of 3 or more on a scale of 0 to 6. The primary outcome endpoint was as follows: (1) stroke or TIA in the same territory within 30 days; (2) stroke or TIA in the same territory beyond 30 days through 6 months; (3) stroke or TIA in the same territory beyond 30 days through 1 year.

## Statistical analyses

Continuous variables were presented as mean ± standard deviation, and continuous variables with non-normal distribution were summarized as medians (interquartile range). Quantitative data were compared using Student’s *t* test or the Mann–Whitney *U* test. Categorical data were presented as numbers with percentages and were analyzed using the chi-squared test or Fisher exact tests. The event rates (stroke or TIA in the same territory within 30 days, beyond 30 days through 6 months, and beyond 30 days through 1 year) were compared between the two groups. Univariate logistic analysis was used to analyze the influence of the two groups on each outcome node. Multivariate logistic regression analysis was used to remove the risk factors associated with outcomes and calculate 95% confidence intervals (CI). PSM was conducted to balance the baseline characteristics between the PTAS and aggressive medical therapy group. Pairs were matched without replacement on the logit of the propensity score, and a nearest-neighbor 1:1 matching scheme with a caliper size of 0.2 was applied for all matched pairs. Bivariate and multivariable logistic analyses were used to compare outcomes. A *p*-value < 0.05 was considered statistically significant. The statistical analyses were performed using the Statistical Package for the Social Sciences (SPSS) version27.0 (SPSS Inc., Chicago, IL, USA).

## Results

**FIGURE 1:**
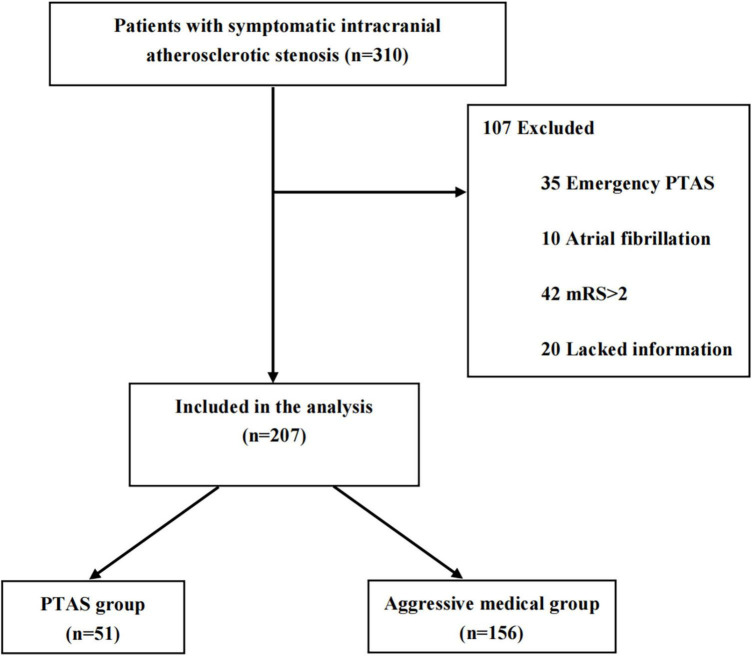
Study flow diagram. PTAS, percutaneous angioplasty and/or stenting; mRS, modified Rankin Scale.

From March 2020 to February 2022, a total of 310 patients with sICAS were included in this study. Among them, 35 received emergency PTAS, 10 had atrial fibrillation, 42 were mRS > 2 and 20 lacked information. A total of 107 ineligible patients were excluded. Finally, 207 patients were enrolled in the study (51 in the PTAS group and 156 in the aggressive medical group) (Figure 1). The baseline characteristics of the patients were nearly balanced between the two groups. The average age of the cohort was 63.8 ± 11.8 years, and 70.5% of patients were male. The stenotic arteries involved the MCA in 133 patients (64.3%), ICA in 38 (18.4%), VA in 21 (10.1%), and BA in 15 (7.2%). As the study population consisted of patients with minor stroke and TIA, the median NIHSS score was 2 (IQR 0–3) (Table 1).

**TABLE 1:** Baseline characteristics of patients.

	Total (*n =* 207)	PTAS group (*n =* 51)	Aggressive medical group (* =* 156)	*p*-value-original cohort	*p*-value-matched cohort
Age(years), mean ± SD	63.8 ± 11.8	61.9 ± 10.5	64.5 ± 12. 1	0. 192	0.589
Sex, male, n (%)	146 (70.5)	38 (74.5)	108 (69.2)	0.473	0.871
BMI, median (IQR) Medical history, n (%)	24.46 (22.83–26.72)	24.22 (22.98–25.95)	24.76 (22.76–27. 16)	0.557	0.917
Hypertension	158 (76.3)	37 (72.5)	121 (77.6)	0.419	0.742
Diabetes	71 (34.3)	18 (35.3)	53 (34.0)	0.909	0.780
Coronal atherosclerosis heart disease	18 (8.7)	2 (3.9)	16 (10.3)	0. 163	0.756
History of stroke or TIA	62 (30.0)	23 (45.1)	39 (25.0)	0.007	0.863
Smoking history, n (%)	88 (42.5)	17 (33.3)	71 (45.5)	0. 127	0.860
Low-density lipoprotein-cholesterol (mg/dL), median (IQR)	2.45 (1.8–3.04)	1.86 (1.41–2.66)	2.56 (1.96–3. 12)	<0.001	0.020
Systolic blood pressure(mmHg), median (IQR)	140 (130–151)	130 (122–140)	140 (130–156)	<0.001	0. 115
Symptomatic qualifying artery, n (%)				0. 158	0.960
Middle cerebral artery	133 (64.3)	26 (51.0)	107 (68.6)		
Intracranial internal carotid artery	38 (18.4)	13 (25.5)	25 (16.0)		
Basilar artery	15 (7.2)	5 (9.8)	10 (6.4)		
Intracranial vertebral artery	21 (10.1)	7 (13.7)	14 (9.0)		
Stenosis of symptomatic qualifying artery, n (%)				0.890	<0.001
70–79	38 (18.4)	9 (17.6)	29 (18.6)		
80–89	74 (35.7)	17 (33.3)	57 (36.5)		
90–99	95 (45.9)	25 (49.0)	70 (44.9)		
Baseline NIHSS score, median (IQR)	2 (0–3)	2 (0–4)	1 (0–3)	0.218	0. 123
Interventions n (%)					
Simple balloon dilation angioplasty		0 (0.0%)			
Self-expanding stenting		1 (2.0%)			
Balloon dilated stenting		50 (98.0%)			

PTAS, percutaneous angioplasty and/or stenting; SD, standard deviation; BMI, body mass index; TIA, transient ischemic attack; IQR, interquartile range; NIHSS, National Institutes of Health Stroke Scale.

The occurrence of primary endpoints in the PTAS group vs. the aggressive medical group was 8/51 (15.7%) vs. 6/156 (3.8%) within 30 days (*P* = 0.003), 1/51 (2.0%) vs. 8/156 (5.1%) beyond 30 days through 6 months (*P* = 0.570), 2/50 (4.0%) vs. 11/155 (7.1%) beyond 30 days through 1 year (*P* = 0.739) (Table 2). The risk of stroke or TIA in the same territory within 30 days was 8/51 (15.7%) vs. 6/156 (3.8%) (*P* = 0.003). No significant difference was found between the groups for the risk of stroke or TIA in the same territory beyond 30 days through 6 months and beyond 30 days through 1 year. Intracranial hemorrhage occurred in 1 patient (0.6%) in the aggressive medical group and none in the PTAS group (*P* = 1.000). There was no significant difference in the rate of 12-month risk of death (*P* = 0.990). For the incidence of disabling stroke (mRS > 2) within 1 year, 9 patients (18.0%) were in the PTAS group and 16 patients (10.3%) were in the aggressive medical group (*P* = 0.149). In multivariate logistic regression, adjusting for age, sex, SBS, LDL-C, and the history of stroke or TIA, there was still no significant difference between the PTAS and aggressive medical groups for clinical outcomes except for the recurrence of stroke or TIA in the same territory within 30 days (*P* = 0.010). Subsequently, PSM analysis was performed to balance the baseline characteristics between the PTAS and aggressive medical groups. The results showed that PSM yielded 88 patients with sICAS who underwent PTAS or aggressive medical therapy. After PSM, there was no significant difference in all outcomes (*P* > 0.05, Table 3). For the PTAS group, all patients underwent balloon dilated stenting (BMS) except for one of the 51 patients who received self-expanding stenting (SES) (Supplementary Table 1). Due to the small number of cases, subgroup analysis could not be conducted.

**TABLE 2:** Primary and secondary outcomes.

	PTAS group (*n* = 51) (%)	Aggressive medical group (*n* = 156) (%)	Risk estimate (95% CI)	*p* value	Adjusted OR (95% CI)	*p* value
Stroke or TIA in the same territory within 30 d	8/51 (15.7%)	6/156 (3.8%)	4.651 (1.531–14.134)	0.003	0.179 (0.048–0.659)	0.010
Stroke or TIA in the same territory beyond 30 d through 6 m	1/51 (2.0%)	8/156 (5.1%)	0.370 (0.045–3.032)	0.570	2.051 (0.232–18.112)	0.518
Stroke or TIA in the same territory beyond 30 d through 1 y	2/50 (4.0%)	11/155 (7.1%)	0.117 (0.545–2.549)	0.739	1.509 (0.301–7.580)	0.617
Disabling stroke within 1 y (mRS > 2)	9/50 (18.0%)	16/155 (10.3%)	1.907 (0.785–4.634)	0.149	0.453 (0.161–1.274)	0.133
Death within 1 y	1/51 (2.0%)	1/156 (0.6%)	3.100 (0.190–50.475)	0.990	0.491 (0.021–11.299)	0.657
Intracranial hemorrhage	0/50 (0.0%)	1/155 (0.6%)		1.000		0.997

Adjusting for age, sex, SBS, LDL-C and the history of stroke or TIA; PTAS, percutaneous angioplasty and/or stenting; TIA, transient ischemic attack; OR, odds ratio; CI, confidence intervals; mRS, modified Rankin Scale; SBS, systolic blood pressure; LDL-C, low-density lipoprotein-cholesterol.

**TABLE 3:** Primary and secondary outcomes after PSM.

	Crude OR (95% CI)	*p* value-matched cohort	Adjusted OR (95% CI)	*p* value-matched cohort
Stroke or TIA in the same territory within 30 d	0.307 (0.061–1.540)	0.151	0.334 (0.059–1.880)	0.214
Stroke or TIA in the same territory beyond 30 d through 6 m	1.389 (0.084–22.948)	0.818	2.711 (0.095–77.524)	0.560
Stroke or TIA in the same territory beyond 30 d through 1 y	0.667 (0.058–7.641)	0.745	0.900 (0.065–12.571)	0.938
Disabling stroke within 1 y (mRS > 2)	0.402 (0.101–1.603)	0.197	0.494 (0.096–2.552)	0.400
Death within 1 y	0	0.998	0	0.998
Intracranial hemorrhage				

Adjusting for age, sex, SBS, LDL-C and the history of stroke or TIA; PSM, propensity score matching; TIA, transient ischemic attack; OR, odds ratio; CI, confidence intervals; mRS, modified Rankin Scale; SBS, systolic blood pressure; LDL-C, low-density lipoprotein-cholesterol.

## Discussion

This observational prospective study showed no significant difference in stroke or TIA risk over 30 days to 1 year in the same territory after PTAS compared to aggressive medical therapy. There was also no significant difference for secondary outcomes.

However, a significant difference was observed in the occurrence of ischemic events within 30 days (*P* = 0.003). This finding is consistent with the SAMMPRIS (Derdeyn et al., 2014) and VISSIT (Zaidat et al., 2015) trials, which demonstrated that stenting carries a higher risk than medication for sICAS patients. At day 30, the incidence of the primary endpoint (any stroke or death) was 14.7% vs. 5.8% (*P* = 0.0016) and 24.1% vs. 9.4% (*P* = 0.050) in the stenting and medical groups, respectively. This difference may be attributed to the perioperative risk of surgery (Turan et al., 2022), the timing of stenting (Derdeyn et al., 2014; Zaidat et al., 2015; Gao et al., 2022) and difference in stent selection, such as BMS, SES, and simple balloon dilation angioplasty (BDA) (Mao et al., 2022; Ueda et al., 2022) as well as the plaque detachment or reperfusion injury (Yaghi et al., 2019). The CASSISS (Gao et al., 2022) trial demonstrated that minimizing perioperative complications did not result in a significant difference in the risk of stroke within 30 days or beyond 30 days to 1 year (8.0% vs. 7.2%, *P* = 0.820). Meanwhile, after PSM analysis, no significant difference was also observed for any stroke risk between these two groups.

Mounting evidence indicates that most recurrent cerebral ischemic events occur shortly after the initial ischemic event, and early stent implantation may increase surgical risks (Zhang et al., 2020). Multiple studies (Kasner et al., 2006; Yaghi et al., 2019; Zhang et al., 2020) have shown that endovascular therapy at least 3 weeks after the initial event appeared to be safer than that at less than 3 weeks. When endovascular treatment was performed over 3 weeks, the incidence of short-term death or stroke was 4.3% and 2%, respectively (Miao et al., 2015a; Wang et al., 2020). The CASSISS trial (median time, 35 days) (Gao et al., 2022), WEAVE trial (median time, 22 days) (Alexander et al., 2019) and a retrospective study trial (median time, 21 days) (Mao et al., 2022) had significantly longer interval from onset to endovascular treatment than the SAMMPRIS trial (median time, 7 days) (Derdeyn et al., 2014) and VISSIT trial (median time, 9 days) (Zaidat et al., 2015), thus avoiding the highest risk period of stroke recurrence. This approach also reduced the incidence of perioperative complications after stenting.

Despite the use of aggressive medical therapy, some patients remain at a high risk for stroke, particularly those with impaired hemodynamics (Amin-Hanjani et al., 2016; Wabnitz et al., 2018). sICAS is believed to be associated with plaque rupture, intraplaque hemorrhage, and thrombosis through various mechanisms such as impaired distal perfusion, arterial-arterial embolism, or perforator disease (Li et al., 2023). Aggressive medical therapy is more effective for patients whose only mechanism is arterial-arterial embolism or perforator disease than for patients whose mechanism is distal perfusion impairment (Bodle et al., 2013; Yaghi et al., 2019). In patients with distal flow impairment, medical therapy may help stabilize atherosclerotic plaques and intracranial thrombi, and reduce the risk of worsening embolism and stenosis, but it is unlikely to rapidly improve flow tissue that is at risk of early recurrence and may benefit from revascularization. Recurrent stroke is linked to no improvement in cerebral perfusion during the perioperative period (Yan et al., 2022). The importance of intracranial hemodynamic impairment as a risk factor for stroke has been demonstrated in both the carotid (Wabnitz et al., 2018) and posterior (Maier et al., 2018) circulation. Although many studies have suggested that stenting is less beneficial than medical therapy alone, these studies have not stratified patients by perfusion status. As such, exploring the safety and feasibility of stenting in high-risk patients with impaired blood flow in sICAS is necessary (Stapleton et al., 2020). The primary endpoint of the sICAS primary treatment trial was focused on clinical stroke or TIA, while subclinical infarction was often omitted from the primary analysis (Moftakhar et al., 2005; Levine et al., 2015).

Currently, there is no consensus on the optimal endovascular treatment strategy for sICAS. Nonetheless, previous studies and our study have shown no significant difference between PTAS and aggressive medical therapy. Randomized controlled trials should remain the gold standard to guide treatment, but biomarkers that predict the risk of recurrent stroke may be used to aid in clinical decision-making and lead to more targeted and personalized treatment (Lee et al., 2011; Ballout and Liebeskind, 2022). Nowadays, with the development of endovascular therapy, there are many interventions, such as drug-coated balloons, drug-eluting stents and other self-expanding stents, or combinations (Gruber et al., 2018, 2019; Han et al., 2019; Kadooka et al., 2020; Remonda et al., 2021; Jia et al., 2022; Si et al., 2022). In order to select the best treatment, the location of the narrowed artery, lesion shape, and lesion pathway must be considered. A comprehensive risk assessment of the treatment procedure should be performed, taking into account other risk factors such as the patient’s lifestyle, clinical factors, and the time interval between the final ischemic attacks (Ueda et al., 2022). Future studies should aim to establish clinical, serological, and imaging biomarkers that can identify high-risk patients (Banerjee and Chimowitz, 2017).

It was a single-center observational study that might have been influenced by selection bias. However, the results of our study provided valuable insights and need to be further confirmed by multicenter large-sample randomized controlled studies that reduce the effect of individual selection on the equivalence of results and enhance the generalizability of the findings. Apart from the crude comparison of the baseline disease events in the anterior vs. posterior circulation, our analysis did not evaluate submaximal angioplasty by specific lesion location and the degree of difference in stenosis before and after intervention. Additionally, the effectiveness of antiplatelet drugs was not supported by the test results in our study, and future studies need to compensate for the influence of drug selection such as aspirin and clopidogrel. Our study was limited to the inclusion of BMS treatment and did not evaluate other types of interventions mentioned above. We used routine medical history, NIHSS, and mRS scores at follow-up without systematic brain imaging to identify high-risk potential recurrent strokes, such as hypoperfusion, and a vascular remodeling index. Future studies should aim to overcome these limitations and provide more comprehensive and detailed analyses to improve patient care and outcomes.

## Conclusion

For patients with TIA or ischemic stroke caused by symptomatic severe intracranial atherosclerotic stenosis, PTAS had a similar effect on preventing stroke or TIA risk as aggressive medical therapy within 1 year compared to the latter. Future RCT trials should be performed to confirm these findings.

## Data availability statement

The original contributions presented in this study are included in the article/Supplementary material, further inquiries can be directed to the corresponding authors.

## Ethics statement

The studies involving human participants were reviewed and approved by Ethics Committee of Nanjing Medical University [No. (2019)695]. The patients/participants provided their written informed consent to participate in this study.

## Author contributions

XL, QD, and JZ contributed to conceptualization. XL, XQ, CL, and HSun contributed to formal analysis and the first draft of the manuscript. All authors made substantial contribution to the design, implementation of the study, reviewed, and approved the final manuscript.

## Footnotes

1
www.clinicaltrials.gov
