# Supplementary material for: Percutaneous angioplasty and/or stenting versus aggressive medical therapy in patients with symptomatic intracranial atherosclerotic stenosis: a 1-year follow-up study
Source: Front Aging Neurosci. 2023 Jun 16;15:1192681. doi: 10.3389/fnagi.2023.1192681 (PMC10313453; doi:10.3389/fnagi.2023.1192681)
Supplement: Supplementary file 1 [file Table_1.docx]

**Table S1：Case prognosis**

| Interventions | Event (N=51) | Stroke or TIA in the same territory | | | mRs>2 within 1y (N=9) | Death within 1y | Intracranial hemorrhage |
| --- | --- | --- | --- | --- | --- | --- | --- |
|  |  | Within 30 d | beyond 30 d through 6m | beyond 30 d within 1 y |  |  |  |
| self-expanding stents | 1 | No | No | No | No | No | No |

Abbreviations: TIA, transient ischemic attack; mRS, modified Rankin Scale.
